# Ultrasensitive Electrochemical Aptasensing of Malathion Based on Hydroxylated Black Phosphorus/Poly-L-Lysine Composite

**DOI:** 10.3390/bios13070735

**Published:** 2023-07-16

**Authors:** Tingting Ma, Jie Zhou, Dan Wei, Hongquan Peng, Xun Liu, Wenfei Guo, Chuanxiang Zhang, Xueying Liu, Song Li, Yan Deng

**Affiliations:** 1 Hunan Key Laboratory of Biomedical Nanomaterials and Devices, Hunan University of Technology, Zhuzhou 412007, China; mzxmatingting@163.com (T.M.); zj1030467675@163.com (J.Z.); m20077700008@hut.edu.cn (D.W.); guowf3220@126.com (W.G.); cxzhang7335@126.zcx (C.Z.); liuxueying@hut.edu.cn (X.L.); 2 Institute for Future Sciences, University of South, Changsha 410000, China; 3 Hengyang Medical School, University of South, Hengyang 421001, China; 4 Department of Nephrology, Kiang Wu Hospital, Macau SAR, China; hpeng93170@gmail.com; 5 Department of Nephrology, The Third Affiliated Hospital of Sun Yat-Sen University, Guangzhou 510630, China; naturestyle@163.com

**Keywords:** malathion, electrochemical aptasensor, aptamers (apt), Au nanoparticles (AuNPs), hydroxylated black phosphorus/poly-L-lysine (hBP/PLL) composite

## Abstract

A highly sensitive unlabeled electrochemical aptasensor based on hydroxylated black phosphorus/poly-L-lysine (hBP/PLL) composite is introduced herein for the detection of malathion. Poly-L-lysine (PLL) with adhesion and coating properties adhere to the surface of the nanosheets by noncovalent interactions with underlying hydroxylated black phosphorus nanosheets (hBP) to produce the hBP/PLL composite. The as-synthesized hBP/PLL composite bonded to Au nanoparticles (Au NPs) firmly by assembling and using them as a substrate for the aptamer with high specificity as a probe to fabricate the sensor. Under optimal conditions, the linear range of the electrochemical aptasensor was 0.1 pM~1 μM, and the detection limit was 2.805 fM. The electrochemical aptasensor has great selectivity, a low detection limit, and anti-interference, which has potential application prospects in the field of rapid trace detection of pesticide residues.

## 1. Introduction

Malathion (Diethyl 2-[(dimethoxyphosphorothioyl) sulfanyl] butanedioate) is a kind of commonly used organophosphorus pesticide used as an efficient insecticide to control pests in agricultural and residential environments and improve crop yield [1]. Only 1% of pesticides sprayed daily on the surface of crops have a real insecticidal effect, while most of the malathion remains on the ground. The long-time residual malathion in the environment may lead to the formation of highly toxic metabolites with carcinogenic and neurotoxic effects, which are harmful to human health and the environment [2]. China promulgated the national standard for food safety in 2019, which stipulated that the maximum residue limit of malathion in food is 0.3 mg/kg and that in drinking water is 0.05 mg/L. Most of the existing pesticide detection methods are instrumental analyses, such as high-performance liquid chromatography (HPLC), gas chromatography-mass spectrometry (GC-MS), and enzyme-linked immunosorbent assay (ELISA), which have advantages such as sensitive detection and accurate results [3]. However, they also have the disadvantages of requiring large-scale precision detection instruments with a high cost and site limitations. The instruments’ operation needs professionals, and the sample pretreatment process is very tedious and time-consuming [4]. Thus, the application of these instrumental analyses in food detection is greatly limited. With the improvement in public awareness of environmental protection, more and more efficient and low-residue pesticides have been developed and applied in agricultural production, and corresponding pesticide residues have the characteristics of ultra-low concentration residues. Therefore, there is an urgent need to establish a reliable, effective, and rapid method to detect pesticides in the environment and agricultural products. Biosensors have advantages such as high sensitivity, great selectivity, low environmental pollution, miniaturized equipment, and suitability for site detection [5,6]. Biosensors use probes to detect different concentrations of target substances and convert the identification of target substance concentration changes into detectable physical signals [7], such as optical signals, electrical signals [8], or magnetic signals. Because of high sensitivity and selectivity, biosensors can be effectively applied in the detection of ultra-low concentration pesticide residues, and constructed biosensors can be further applied to data acquisition systems and other fields.

As one of the new members of the two-dimensional (2D) nanomaterial family, black phosphorus (BP) has advantages such as high carrier mobility [9], great conductivity [10] and biocompatibility, and optoelectronic and photothermal properties [11,12]. As substrate material for biosensors, it can effectively improve the performance of sensors [13]. Black phosphorus nanosheets have wrinkled surfaces, and their surface area is relatively large [14], which can increase carrier mobility [15] and provide more active sites [16,17]. However, there are two lone pair electrons in each phosphorus atom [18], so it is highly sensitive to oxygen and humidity [19], easily oxidized and degraded by air, and its stability is poor [20]. Aptamers are “chemical antibodies”, which also have some advantages that antibodies do not have, such as easy chemical synthesis [21], non-immunogenicity, small size, good biochemical stability [22], and so on. The targets for aptamers include proteins [23], viruses, small molecular organics [24,25,26,27], and living cells, which can bind to a wide range of substances. Therefore, aptamers are often used as molecular probes to improve the selectivity and specificity of biosensors [28,29,30]. Poly-L-lysine (PLL) is a cationic polymer that can produce strong electrostatic force with anion-containing substances [31,32] and has high acid and alkali resistance, high bacteriostatic activity, and thermal stability [33]. It is also often used as a cationic biological active agent to improve the adhesion of sensor substrate materials in the field of biosensors [12].

In this study, an aptamer electrochemical sensor based on hydroxylated black phosphorus nanoparticles/poly-L-lysine(hBP/PLL) composite was constructed to detect malathion with specificity and high sensitivity. Firstly, we prepared hydroxylated black phosphorus nanosheets(hBP), which were then incubated and coated with the bioactive material poly-L-lysine (PLL) to further improve the environmental stability of black phosphorous. Then, a layer of AuNPs was added to the glassy carbon electrode with hBP/PLL composites, which can amplify the electrochemical signal and act as a bioactive site to combine aptamers [34]. The aptamer is used as a probe to specifically identify and recognize malathion. The constructed electrochemical aptamer sensor has high sensitivity and specificity for malathion and can be used for rapid detection of malathion in the field.

## 2. Materials and Methods

### 2.1. Reagents and Apparatus

Black phosphorus (BP) was obtained from Six Carbon Technology Co., Ltd. (Shenzhen, China). N-methyl-2-pyrrolidone (NMP), Poly-L-lysine (PLL), 20 × PBS buffer, absolute ethanol (CH_3_CH_2_OH), and Tris(2-carboxyethyl) phosphine hydrochloride (TCEP) were purchased from Sangon Biotech Co., Ltd. (Shanghai, China). Potassium ferricyanide (K_3_[Fe (CN)_6_]) and Potassium chloride (KCl) were acquired from Sinophram Chemical Reagents Co., Ltd. (Shanghai, China). Sodium hydroxide (NaOH) and 6-mercapto-1-hexanol (MCH) were purchased from Sigma-Aldrich (St. Louis, MO, USA). Malathion and other pesticides were purchased from Tanmo Quality of Science and Technology Co., Ltd. (Changzhou, China). Nano gold colloid, graphene oxide dispersion(rGO), and graphene dispersion (GO) were purchased from Jiangsu XFNANO Materials Tech. Co., Ltd. (Nanjing, China). The aptamer sequence was 5′SH-ATCCGTCACACCTGCTCTTATACACAATT-GTTTTTCTCTTAACTTCTTGACTGCTGGTGTTGGCTCCCGTAT-3′, which was synthesized through Sangon Biotech Co., Ltd. (Shanghai, China). The aptamer was stored in 1 mM PBS, and oxygen-free water was used for the whole experiment.

The cyclic voltammetry (CV) (with the potential scan range set from −0.2 V to 0.6 V; other parameters are default values) and differential pulse voltammetry (DPV) (with the potential scan range set from −0.2 V to 0.5 V; other parameters are default values) experiments were performed on a PGSTA T302N electrochemical workstation (Metrohm, Switzerland). A conventional three-electrode system included a modified glassy carbon electrode (GCE) (3 mm in diameter) as working electrode, a platinum wire electrode as auxiliary electrode, and a saturated calomel electrode (SCE) as reference electrode. X-ray photoelectron spectroscopy (XPS) was obtained from ESCALAB250Xi (ThermoFisher-VG Scientific, Waltham, MA, USA). Scanning electron microscopy (SEM) and energy dispersive spectroscopy (EDS) were obtained from a Quanta 250 FEG (FEI, USA). The Raman spectroscopy was used through inVia (Renishaw, Wotton-under-Edge, UK).

### 2.2. Preparation of hBP/PLL

A total of 40 mL of N-methylpyrrolidone (NMP) and 0.1 g NaOH were added to a 100 mL flask and strongly stirred at a constant temperature for 30 min, which was left standing to obtain the supernatant, which was the saturated solution of NMP and NaOH. Then, 40 mg black phosphorus crystal was added into a centrifuge tube filled with 40 mL of above solution in argon, which was reacted by ultrasonication for 8 h at 25 °C, and then centrifuged at 3000 rpm for 30 min to obtain the supernatant. This supernatant was centrifuged again at 8000 rpm for 30 min in argon, and the obtained supernatant was hBP dispersion solution. A total of 600 μL of hBP dispersion was centrifuged to remove NMP, and 600 μL of ethanol was added with 400 μL of 2 mg/mL PLL solution after shaking and mixing, and then incubated at 25 °C for 1 h and kept overnight at 4 °C. The hBP/PLL composite was prepared and preserved at low temperatures.

### 2.3. Fabrication of Malathion-Aptamer Electrochemical Sensor

**Scheme 1:**
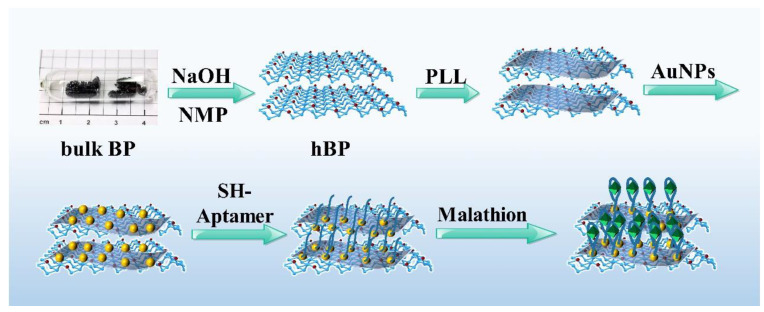
Schematic diagram of constructed hBP/PLL-based aptamer sensing for detection of malathion.

Firstly, AuNPs/hBP/PLL/GCE was prepared by dropping 3 μL of AuNPs onto the GCE modified with hBP/PLL and dried in argon. The 10 μM mercaptosylated aptamer of malathion was prepared at 25 °C and heated at 95 °C for 3 min. The aptamer with chain expansion was obtained by rapid cooling. Then, 10 μM TCEP was added and incubated at 25 °C for 1 h to activate aptamers. A total of 3 μL of activated 10 μM aptamer was dropped onto AuNPs/hBP/PLL/GCE, incubated at 4 °C for 12 h with humidity, and slowly washed with 1 × PBS solution three times. A total of 3 μL of 1 mM MCH solution was dropped and incubated onto the electrode at 25 °C for 1 h, and then slowly washed with 1 × PBS solution three times and dried in argon. Finally, we prepared an aptamer electrochemical sensor (MCH/Apt/AuNPs/hBP/PLL/GCE) and preserved it at 4 °C. Scheme 1 was the principle of malathion detection by aptamer electrochemical sensor. Firstly, hBP was prepared by liquid phase ultrasonic stripping. Stable black phosphorus composite was prepared by combining the hydroxyl group on black phosphorus nanosheets with amino group from PLL. Then, a layer of AuNPs was added onto the hBP/PLL composite as the bioactive site to bind the sulfhydryl groups on the mercaptosylated aptamer, and the current signal was further amplified. After adding the target malathion, due to specific recognition effect of the aptamer, the expanded aptamer was bound with malathion to form a specific secondary structure, resulting in greater steric hindrance and lower DPV peak current. The lower the concentration of malathion was, the less the aptamer binding folded, and the weaker the steric hindrance effect was, which led to increased DPV peak current. Otherwise, the current decreased through this principle to achieve detection of malathion.

### 2.4. Malathion Detection on hBP/PLL-Modified Electrodes

The DPV responses of fabricated aptasensor were recorded in 5 mM [Fe(CN)_6_]^3−/4−^ solution (prepared in 10 mM PBS containing 0.1 M KCl) in the potential window from −0.2 to 0.5 V. Malathion detection was performed according to the following procedure: the obtained MCH/Apt/AuNPs/hBP/PLL/GCE was incubated in 5 mM PBS buffer containing different concentrations of standard malathion solution for 60 min, after which it was washed with 5 mM PBS buffer three times and was then transferred to the electrochemical cell for DPV measurement.

## 3. Results and Discussion

### 3.1. Characterization of hBP/PLL Composite

**Figure 1:**
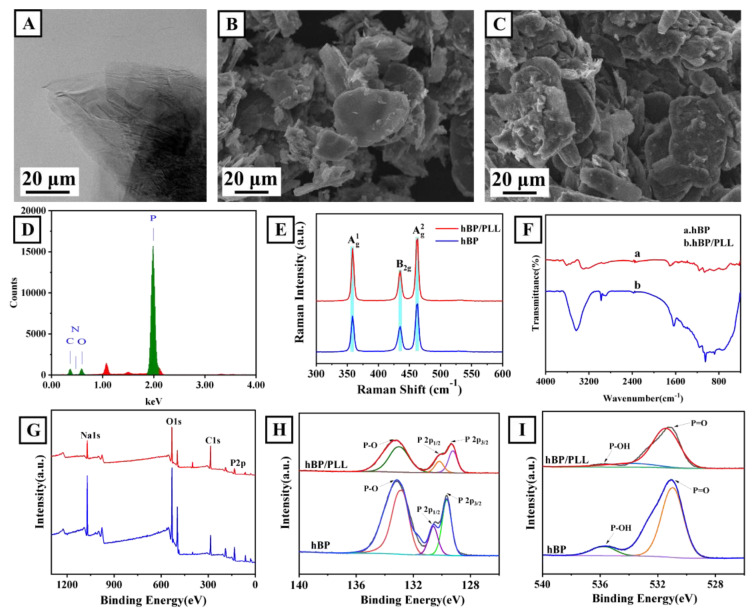
(**A**) TEM image of hBP, (**B**) SEM image of hBP, (**C**) SEM image of hBP/PLL, (**D**) EDS energy spectrum of hBP/PLL, (**E**) Raman spectra for hBP and hBP/PLL composites, (**F**) FTIR spectrum for hBP and hBP/PLL composites, (**G**) XPS spectra for hBP and hBP/PLL composites, (**H**) P 2p core level, and (**I**) O 1 s core level.

Figure 1A,B are TEM and SEM images of hBP after the addition of PLL. The SEM image of hBP/PLL is shown in Figure 1C. The TEM result shows that the prepared hBP had a nano-layered structure. It can be seen from the SEM that the surface morphology of hBP did not change after the addition of PLL. The EDS energy spectra of hBP/PLL (Figure 1D) showed strong peaks of elemental phosphorus and very low peaks of elemental carbon and oxygen. The results can indicate that the hBP/PLL composites are not susceptible to oxidative degradation in aqueous oxygen environment and have good environmental stability.

The Raman spectra for hBP-PLL composite and hBP (Figure 1E) show that the three characteristic Raman peaks for BP were observed at 361.5, 437, and 463.5 cm^−1^, and the three characteristic Raman peaks for phosphorus were not affected by the addition of PLL. These results indicated that the PLL retained the original chemical properties and phase characteristics of hBP. The infrared spectra for hBP and hBP/PLL (Figure 1F) showed enhanced N-H stretching vibration at 3500 cm^−1^ and C-N stretching vibration for PLL at 1200~1025 cm^−1^, indicating that PLL was successfully combined with hBP. The characteristic absorption peak for PLL appeared at 1680~1640 cm^−1^.

Figure 1G is the XPS spectrum for hBP and hBP/PLL. The full scanning spectra for XPS elements of hBP and hBP/PLL showed the peaks for Na, C, O, and P, which were derived from BP and Na_2_CO_3_. The existence valence for the P element in the hBP and hBP/PLL was further analyzed, and the P2p orbital narrow sweep was carried out (Figure 1H). hBP/PLL and hBP both had peaks for unoxidized BP at 129.5 and 130.5 eV, showing that the P-O oxidation peak intensity for hBP at 130.5 eV was significantly reduced after the addition of PLL. In order to further analyze the oxidation of P in the hBP and hBP/PLL, the O1s orbital was narrowly swept (Figure 1I). Moreover, the XPS characteristic peak intensity for P=O at 530.75 eV decreased after the addition of PLL, indicating the degree of hBP oxidation and that PLL had an effective protective effect on hBP. The reduced P-OH peak at 535.8 eV was due to partial binding between the amino group on PLL and the hydroxyl functional group on the hBP, indicating that the PLL was successfully modified on the surface of the hBP, which improved the stability of the hBP to oxygen and environmental stability of the sensor.

### 3.2. Feasibility of the Prepared Biosensor

**Figure 2:**
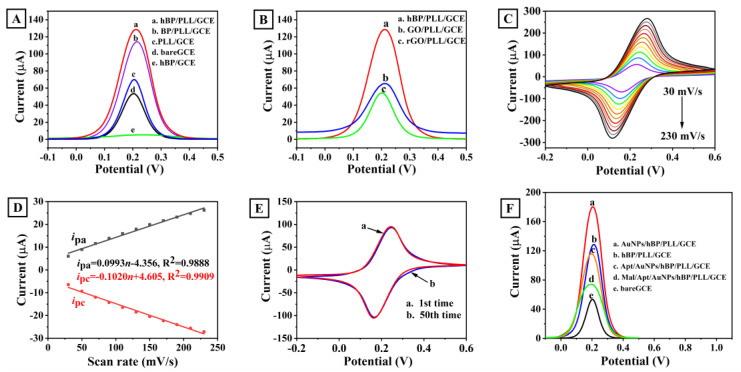
DPV response image of (**A**) hBP/PLL/GCE, BP/PLL/GCE, PLL/GCE, bareGCE, and hBP/GCE, (**B**) DPV response image of hBP/PLL/GCE, GO/PLL/GCE, and rGO/PLL/GCE, (**C**) CV image of hBP/PLL/GCE at different scanning rates from 30 mV/s to 230 mV/s, (**D**) graph of peak current as a function of scan rate, (**E**) CV images of hBP/PLL/GCE scanned for the 1st and 50th, and (**F**) DPV image of step-by-step modification of GCE in the mixture of 5 mM [Fe(CN)_6_]^3−/4−^ + 0.1 m KCl + 10 mM PBS.

First, the electrical conductivity of hBP was investigated. As shown in Figure 2A, the peak current after only dripping with hBP decreased abnormally compared with the bare electrode. This is due to the negative charge of the oxidizing group on BP, which has relatively weak electrostatic repulsion to the redox probe [Fe (CN)_6_]^3−/4−^ with a negative charge. PLL has a certain coating effect on hBP and eliminates the electrostatic repulsion effect. Meanwhile, due to the excellent electrical properties of BP, the peak current value was further improved. Therefore, the peak current for hBP/PLL increased sharply, and the peak current for PLL/GCE was almost similar to the bare electrode, which once again demonstrated good conductivity of hBP. Comparing hBP/PLL with BP/PLL, the hBP/PLL showed a higher DPV peak current, indicating that the conductivity of the prepared hBP was still better than that of the unmodified BP after coating with PLL.

Cyclic voltammetry (CV) tests were carried out on the prepared hBP/PLL/GCE at scanning rates of 30, 50, 70, 90, 110, 130, 150, 170, 190, 210, and 230 mV/s, respectively (Figure 2C). Figure 2D shows that the current values of the oxidation and reduction peaks for hBP/PLL/GCE are linear with CV scanning rate, indicating that the direct electron transfer on hBP/PLL corresponds to the surface control process. The electrochemical stability of hBP/PLL/GCE was investigated by consecutively cyclic scanning (50 cycles) at the scanning rate of 50 mV/s. Figure 2E shows that the two curves for the first scan and the fiftieth scan basically overlap, indicating that the GCE modified by hBP/PLL shows good stability.

Figure 2F is the step-by-step modification diagram of the constructed malathion aptamer electrochemical sensor. DPV peak current shows corresponding changes after drip-coating of different materials, proving the feasibility of the construction of the sensor. Compared with the bare electrode, the peak current for DPV was significantly increased after dropping hBP/PLL composite due to the excellent conductivity of hBP and coating of the PLL. After adding AuNPs, the electrical signals of the sensor were further amplified due to the high electron density and great electrical conductivity of AuNPs, while the AuNPs further connected the mercaptosylated aptamer by the Au-S bond, enhancing the adhesion strength of the electrode and aptamer, effectively preventing the aptamer from falling off during testing. Because the aptamer is a DNA biomacromolecule with poor electrical conductivity, the DPV peak current for Apt/AuNPs/PLL/hBP/GCE decreased. Finally, when Apt/AuNPs/PLL/hBP/GCE is used to detect malathion, the expanded aptamer will bind to malathion to form a specific secondary structure due to the specific recognition of the aptamer, leading to an increase in the potential resistance on the electrode surface and a decrease in the DPV peak current. The higher the concentration of malathion, the more the aptamer binds and folds and the stronger the potential resistance effect, leading to a decrease in the DPV peak current. By this principle, we can achieve the detection of malathion.

### 3.3. Optimization of Incubation Time

**Figure 3:**
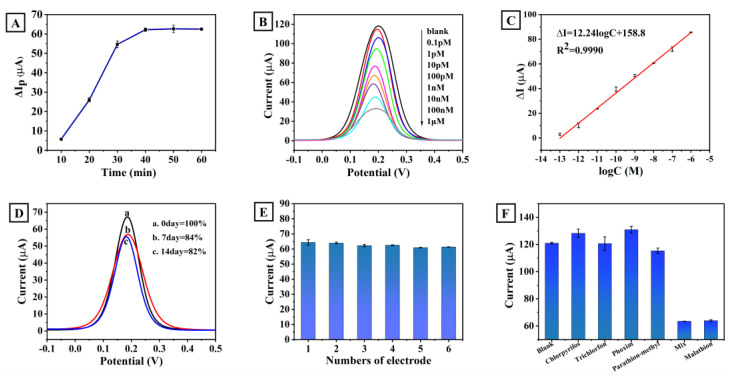
(**A**) Effect of incubation time of aptamer and malathion on DPV peak current of the sensor, (**B**) DPV response image of the sensor to different concentrations of malathion, (**C**) linear image of the relationship between logarithm of malathion concentration and current peak difference, (**D**) DPV current of the sensor after 0, 7, and 14 days, respectively, (**E**) repeatability at six different electrodes in malathion of 1 nM, and (**F**) DPV current of sensors in different pesticides in 5 mM [Fe (CN)_6_]^3−/4−^ + 0.1 m KCl + 10 mM PBS.

The incubation time of the aptamer and malathion was optimized, and the DPV current changes in the aptamer and malathion were investigated for 10, 20, 30, 40, 50, and 60 min. Figure 3A shows that more malathion molecules were bound to the aptamer as the incubation time was increased, and the DPV peak current changed accordingly. When the incubation time exceeded 40 min, the DPV peak current no longer changed, indicating that the binding of the aptamer to malathion had reached a saturated state. Therefore, the optimal incubation time for the aptamer and malathion was 40 min. Further research work will be performed with this optimal incubation time.

### 3.4. Electrochemical Response and Calibration Curves

Using the constructed aptamer electrochemical sensor as the working electrode, the platinum wire electrode as the counter electrode, and the calomel electrode as the reference electrode, a series of malathion standard concentrations were exposed to the aptamer electrochemical sensor, and the DPV test was tested in 5 mM [Fe (CN)_6_]^3−/4−^ + 0.1 M KCl + 10 mM PBS solution with a potential scan range of −0.1~0.5 V for detection of malathion. The resulting DPV presented a concentration-dependent peak, as shown in Figure 3B. It shows that the DPV detection current decreased with increased concentration of malathion due to the effect of spatial conformation. The DPV peak current difference was measured by the aptamer sensor in the concentration range of 0.1 pM~1 μM, which was linearly correlated with the concentration of malathion (Figure 3C). The linear equation was Ip(μA) = 12.24logC + 158.8, the linear correlation coefficient was R^2^ = 0.9990, and the detection limit was 2.805 fM.

As summarized in Table 1, the analytical properties of the aptamer sensor to detect malathion are comparable to or even exceed those of previously reported aptamer sensors.

### 3.5. Selectivity, Stability, and Reproducibility of the Proposed Aptasensor

Figure 3D is the DPV current diagram for the aptamer electrochemical sensor after being placed for 0, 7, and 14 days, respectively. As can be seen from the figure, the current decreased by 16% after 7 days, but the current signal was basically stable after that (decreasing by 2%), indicating that the sensor has great stability. The parallel tests of six different electrodes (Figure 3E) showed that the DPV peak current signals of different electrodes for the same concentration of malathion were basically consistent, which indicated that the constructed aptamer electrochemical sensor has good repeatability.

The selectivity of the aptamer electrochemical sensor was evaluated with blank control, 10 nM chlorpyrifos, trichlorfon, phoxim, parathion-methyl, 1 nM malathion, and five pesticides of mixtures. The test results are shown in Figure 3F. Even though the concentrations of other interfering pesticides except malathion were higher, their DPV peak current response values were basically the same as those of the blank sample. When malathion was added, the DPV peak current response decreased significantly, which was attributed to the high specificity of the aptamer. The aptamer is the probe of the sensor, only recognized and folded into a specific secondary structure for malathion. The experimental result showed that the aptamer electrochemical sensor had great selectivity.

### 3.6. Practical Application

In order to test the application of the aptamer electrochemical sensor in practical samples, the standard addition method was used to detect different concentrations of malathion in lake water, soil samples, greengrocery, and cabbage, and the recovery rate was calculated. Through analysis of Table 2, the results showed that the recovery range was 92.6% to 96.6%, indicating that the sensor is expected to be applied in the detection of malathion in actual samples.

## 4. Conclusions

In this study, a novel aptamer electrochemical sensor was developed for the detection of malathion in real samples. The sensor used the adhesion and coating property of PLL to prepare hBP/PLL composite with great environmental stability and used hBP/PLL composite and AuNPs as the sensor substrate for electrical signal amplification. Using the aptamer with high specificity as the probe, the sensor has advantages such as a low detection limit, high selectivity, great reproducibility, and stability. The linear range of the sensor is 0.1 pM~1 μM, and the detection limit is 2.805 fM. The aptamer sensor provides a new method for the rapid detection of malathion in real places and has a potential application prospect in the field of rapid trace detection of pesticide residues.

## Figures and Tables

**Table 1:** A comparison of the Apt/AuNPs/PLL/hBP/GCE on detecting malathion with previously reported electrodes.

Electrode	Method	Linear Range/μM	LOD/μM	Ref.
Carbon paste electrode	Electrochemical sensor	6.0 × 10^−4^–2.4 × 10^−2^	1.7 × 10^−4^	[35]
Graphite electrode	Molecularly imprinted sensor	3.9 × 10^−5^–3.9	4.2 × 10^−6^	[36]
Screen-printed gold electrode	Molecularly imprinted sensor	3.0 × 10^−7^–3 × 10^−3^	1.8 × 10^−7^	[37]
Glassy carbon electrode	Electrochemical sensor	3.0 × 10^−5^–1.5 × 10^−3^	1.0 × 10^−5^	[38]
Glassy carbon electrode	Aptamer-based electrochemical biosensor	7.6 × 10^−5^–2.6 × 10^−3^	5.2 × 10^−5^	[39]
Glassy carbon electrode	Electrochemical sensor	1.5–61.0	2.4 × 10^−3^	[40]
Glassy carbon electrode	Electrochemical sensor	3.0 × 10^−5^–3.0 × 10^−3^	1.0 × 10^−5^	[41]
Pencil graphite electrodes	Electrochemical sensor	8.9 × 10^−4^–4.5 × 10^−2^	6.8 × 10^−4^	[42]
Glassy carbon electrode	Aptamer-based electrochemical biosensor	1.0 × 10^−7^–1.0	2.8 × 10^−9^	This work

**Table 2:** Determination of malathion in real samples.

Sample	Add (nM)	Found (nM)	Recovery (%)
Lake water-1	0.1	0.09356	93.56
Lake water-2	1	0.9265	92.65
Soil-1	0.1	0.09658	96.58
Soil-2	1	0.9544	95.44
Greengrocery-1	0.1	0.09363	93.63
Greengrocery-2	1	0.931	93.10
Cabbage-1	0.1	0.09513	95.13
Cabbage-2	1	0.9544	95.44

## Data Availability

Not applicable.
